# Viral vector-based influenza vaccines

**DOI:** 10.1080/21645515.2016.1210729

**Published:** 2016-07-25

**Authors:** Rory D. de Vries, Guus F. Rimmelzwaan

**Affiliations:** Department of Viroscience, Erasmus MC, Rotterdam, The Netherlands

**Keywords:** adenovirus, immunity, influenza, MVA, vector, vaccine

## Abstract

Antigenic drift of seasonal influenza viruses and the occasional introduction of influenza viruses of novel subtypes into the human population complicate the timely production of effective vaccines that antigenically match the virus strains that cause epidemic or pandemic outbreaks. The development of game-changing vaccines that induce broadly protective immunity against a wide variety of influenza viruses is an unmet need, in which recombinant viral vectors may provide. Use of viral vectors allows the delivery of any influenza virus antigen, or derivative thereof, to the immune system, resulting in the optimal induction of virus-specific B- and T-cell responses against this antigen of choice. This systematic review discusses results obtained with vectored influenza virus vaccines and advantages and disadvantages of the currently available viral vectors.

## Introduction

Influenza viruses belong to the family of *Orthomyxoviridae*, are an important cause of acute respiratory infections and cause annual epidemics in the human population. Although in most cases infections are self-limiting and restricted to the upper respiratory tract, certain patient groups (such as the elderly) are at risk of developing complications leading to high morbidity and mortality. Vaccines against circulating influenza strains are readily available and are trivalent or quadrivalent, designed to protect against influenza viruses of both the A(H1N1) and A(H3N2) subtype, and against one or both lineages of influenza B virus.

Several different vaccine formulations are available: trivalent or quadrivalent inactivated virus vaccines (TIV or QIV, either whole virus, split virus or subunit vaccines) or live attenuated influenza virus vaccines (LAIV). Most vaccines are produced in embryonated chicken eggs, but vaccines produced in mammalian or insect cells are also available. Inactivated vaccines are administered intramuscularly (IM) or sometimes intradermally and predominantly aim at the induction of serum antibody responses against the viral hemagglutinin (HA) and neuraminidase (NA) to a lesser extent. Protection from disease is mainly mediated by virus neutralizing antibodies against HA, but NA-specific antibodies also contribute to protective immunity.^1^ Currently licensed LAIV are administered locally via nasal spray. Viruses are attenuated by the choice of a viral backbone of cold-adapted viruses and are therefore temperature-sensitive and replicate only locally after administration at the mucosa of the nasopharynx.^2^ In addition to serum antibodies, immunization with LAIV also induces mucosal antibodies and cytotoxic T-lymphocytes (CTL).

Although currently available influenza vaccines are effective in reducing morbidity and mortality caused by seasonal influenza viruses, they have several limitations. Mainly, continuous antigenic drift of seasonal influenza viruses complicates the production of effective vaccines. The vaccine strains need to be updated almost annually in order to achieve a good antigenic match with the epidemic virus strains. If the vaccine strains do not antigenically match the circulating strains, vaccine efficacy is reduced considerably, as was the case in the 2014-2015 influenza season.^3-5^ Furthermore, the seasonal influenza vaccines will afford little or no protection against antigenically distinct pandemic influenza viruses, which are often of alternative subtypes to which antibodies are virtually absent in the human population. During the last decades zoonotic transmissions of highly pathogenic avian influenza viruses, in particular those of the H5N1 subtype, have been reported regularly. The capacity of A(H5N1) and avian viruses of other subtypes including A(H5N6),^6^ A(H7N7),^7^ A(H7N9),^8^ A(H9N2)^9^ and A(H10N8)^10^ to infect humans fuelled the fear for a pandemic outbreak caused by any of these viruses.

H5N1 vaccines that were produced according the procedures used for the production of seasonal influenza vaccines proved to be poorly immunogenic and in most cases the use of adjuvants was required for the efficient induction of protective antibody levels.^11^ Furthermore, the pandemic of 2009 caused by swine-origin influenza viruses of the A(H1N1) subtype (H1N1pdm09) taught an important lesson. The production of tailor made pandemic influenza vaccine proved to be a time-consuming process and in many countries vaccines became available after the peak of the pandemic.^12^

These limitations of the currently available vaccine production technologies and vaccines underscore the pressing need for game-changing vaccines. In addition to improving immunogenicity in the high risk groups, novel vaccines are required that induce long-lasting immunity against a wide range of influenza viruses and that can be produced rapidly in the face of a pandemic outbreak. To improve immunogenicity of influenza vaccines specifically in the elderly, high-dose vaccines and an adjuvanted vaccine have been developed. The latter has been in use in Europe and the US since 1997 and 2015, respectively.

The use of viral vectors for influenza vaccine production may be a solution to some of the problems discussed above. In this review we discuss various viral vectors that have been tested as candidate influenza vaccines in animal models and in clinical trials. Most viral vectors are considered live vaccines but their replication is attenuated or even deficient. Therefore, vector-based vaccines are considered safe in general and some of them can even be safely used in immunocompromised. Despite their attenuated phenotype, viral vectors are immunogenic and induce virus-specific antibody and T cell responses after systemic or parenteral administration. Additionally, most viral vectors can easily be propagated to high virus titers and it is relatively easy to insert genes encoding antigens of choice into the vector. Viral vector technology also allows the production of modified influenza viral antigens *in vivo*. These modifications can improve the immunogenicity of the influenza viral proteins or alter the specificity of the immune response. In this review, we discuss reports on vectored influenza vaccines and discuss their advantages and disadvantages.

## Vectored influenza vaccines

### Pox virus vectors

Smallpox, caused by variola virus, was the first viral disease that was widely prevented and eradicated by vaccination. Originally, Edward Jenner was able to prevent experimental smallpox infection of humans by priming the immune system with the closely related cowpox virus. Vaccinia virus (VV), closely related to the causative agent of cowpox, was thereafter used as one of the vaccines to eradicate smallpox. Since VV has optimal properties to be used as a viral vector, soon after its initial use as cloning vector in 1982^13,14^ VV was used as a vaccine vector to express influenza virus antigens. Smith *et al* were the first to generate VV expressing the influenza HA gene and this vaccine was able to induce an antibody response in rabbits and could protect hamsters from lethal challenge.^15^ Since then, recombinant VV were designed that express all influenza virus proteins.^16^ Although VV vectors expressing influenza antigens were capable of inducing protective immune responses in various animal models, substantial reactogenicity of this vector was frequently observed, which has been addressed by the use of further attenuated and/or replication-deficient strains of VV. An overview of poxvirus-based influenza vaccines can be found in Table 1.

**Table 1.** Overview of poxvirus-based influenza vaccines.

Vector	Model	Antigen	Modification	Subtype	Reference
					
Poxviruses		HA	n/a	H1N1, H5N1	^37,38,43-45,68^
		HA	HAstem	H5N1	^51^
		HA	Mosaic	H5	^63^
		NP	n/a	H5N1	^51^
		M1	n/a	H5N1	^51^
		M2	n/a	H5N1	^51^
		PB2	n/a	H5N1	^51^
		NA	n/a	H5N1	^38,67^
		HA/NP	n/a, HAstem	H1N1, H5N1	^36,51-53,68^
		NP/M1	n/a	H3N2	^56,57^
		HA/NA	n/a	H5N1	^54,68^
		HA/M2	HAstem, M2e repeats	H1N1, H5N1, H7N2, H9N2	^51^
		HA/NP/M2	HAstem, M2e repeats	H1N1, H5N1, H7N2, H9N2	^51^
		HA/NP/NA/M1/M2	n/a	H5N1	^54^
					
		HA	n/a	H1N1, H7N9	^39,50^
					
		HA	n/a	H5N1	^66^
		NP/M1	n/a	H3N2	^57^
					
		HA	n/a	H1N1, H5, H5N1, H5N8, H7N2, H7N7, H7N9, H9N2	^26,77-81,83,84,89-93^
		NP	n/a	?	^93^
		HA/NP	n/a	H5	^93^
		HA/NA	n/a	H5N1	^94,95^
		NP/M1	n/a	H3N2	^55,57^
					
		HA	n/a	H5, H5N1	^81,85,86,88^
					
		HA	n/a	?	^77^
					
		HA	n/a	H3N8	^96^
					
		HA	n/a	H3N8	^49,69-74^
		NP	n/a	H3N8	^49^
					
		HA	n/a	H2N2	^15^
					
		HA	n/a	H2N2	^15^
					
		HA	n/a	H5N1, H5N8	^79,96^
					
		HA	n/a	H1N1, H5N1	^40,46^
		NP	n/a	H5N1	^40^
		HA/NP	n/a	H1N1, H5N1	^40^
					
		HA	n/a	H5N1	^47,48^
		NP/M1	n/a	H3N2	^58-62^

#### Modified vaccinia virus Ankara (MVA) vectors

MVA, an attenuated VV strain, is derived from chorioallantois vaccinia virus Ankara through serial passaging in chicken embryo fibroblasts,^17,18^ resulting in major deletions in the viral genome that influenced many virulence and immune evasion factors.^19-21^ Consequently, MVA replication is highly restricted to avian cells and MVA is unable to produce infectious progeny in most mammalian cell types.^22^ Since MVA is replication-deficient in mammalian cells and therefore lowly reactogenic in humans, it is an attractive vector for vaccination purposes. This was demonstrated in field trials, where MVA was successfully used as a safe smallpox vaccine in over 120,000 individuals in the absence of any serious adverse events.^23^

However, the use of MVA as a vaccine vector has multiple alternative advantages. Notably, MVA safety was confirmed in various *in vivo* models, including avian species and mammals with immunodeficiencies,^24-27^ leading to classification of MVA as a biosafety level 1 (BSL-1) pathogen. Additional advantages of MVA as a vaccine vector include: easy insertion of antigens of interest into the viral genome, transient expression of heterologous antigens *in vivo* and induction of both humoral and cellular responses in animal models and humans. Finally, an interesting characteristic of MVA is that compared to VV, MVA has lost the capacity to evade the host innate immune system.^28-35^ Consequently, vaccination with MVA has an intrinsic immunostimulatory activity (potentially comparable to adjuvants used in combination with vaccination) that leads to rapid influx of various types of immune cells.^34^ Although a potential negative effect of pre-existing vector immunity on immunogenicity is always a concern with the use of vectored vaccines, this does not seem to be a major problem with MVA-based vaccines. It has been shown in humans that a second booster vaccination with a MVA expressing an influenza virus HA, still resulted in potent antibody responses against the protein of interest. Similar observations were made in other studies with MVA expressing other proteins (reviewed in ^36^). This indicates that recombinant MVA remain immunogenic, despite vector immunity.

MVA holds great promise as a vaccine vector and was initially shown to be a promising influenza vaccine in 1994 by Sutter *et al*.^37^ This vaccine was engineered to express the HA and nucleoprotein (NP) gene from influenza virus A/PR/8/34. In addition, recombinant MVA expressing other proteins from various influenza strains were generated and tested in animal models.

#### MVA-HA vaccines

To induce sterile immunity against influenza viruses, HA is the surface antigen of choice since it efficiently stimulates B-cell responses and the production of virus neutralizing antibodies (VN) *in vivo*. Therefore, MVA vector vaccines expressing HA of various subtypes have been constructed and tested in animal models. It should be noted however that most antibodies directed to HA are strain-specific and display poor cross-reactivity with HAs of alternative subtypes, or even with HA molecules from other viruses of the same subtype. Therefore, MVA-HA vaccines often offer protection from infection with the homologous influenza A virus, but not or poorly against infection with heterologous viruses.

Recombinant MVAs expressing the HA gene of the H1N1pdm09 influenza virus A/California/07/09 have been tested for immunogenicity in mice, ferrets and macaques. In different studies, mice could be fully protected from disease after challenge infection with the homologous virus and protection correlated with the induction of VN antibody and T-cell responses.^38,39^ In addition, intra-subtypic cross-immunity was observed to some extent as MVA-H1(A/Cal/07/09) could also protect mice from infection with various A(H1N1) swine influenza viruses.^38^ Immunization of ferrets with a similar recombinant MVA induced robust antibody responses and partially protected from challenge infection with an antigenically closely related H1N1pdm09 virus, A/NL/602/09.^40^ Macaques were also fully protected from H1N1pdm09 virus infection (A/Norway/3487/09) by 2 immunizations with a MVA-based H1N1pdm09 vaccine.^41^ Taken together, these data indicate that an MVA-based vaccine is able to induce protective immunity against the virus that caused the influenza pandemic in 2009, but the extent of cross-protection against unrelated H1N1 viruses is limited.

Highly pathogenic avian influenza viruses of the H5N1 subtype cause endemic outbreaks in poultry. However, since 1997, zoonotic transmission of various A(H5) viruses have caused many cases of human infection^6,42^ of which almost 400 had a fatal outcome. The zoonotic transmission of A(H5N1) viruses, their continuous circulation in wild and domestic birds and the fact that only a few amino acid substitutions are necessary to confer transmissibility between mammals,^43^ underscore the pandemic potential of these viruses. The circulation of A(H5N1) viruses belonging to antigenically distinct clades has complicated vaccine development and has necessitated the selection of various prototypic vaccine strains. Recombinant MVA vaccines expressing HA genes derived from various A(H5N1) strains have been constructed and tested for protective efficacy against viruses belonging to different clades in various animal models. Vaccination with MVA expressing the HA gene of influenza virus A/Vietnam/1194/04 (clade 1) completely protected mice and chickens from infection with the homologous virus and also offered mice protection against infection with influenza viruses A/HK/156/97 (clade 0) and A/Indonesia/5/05 (clade 2.1).^26,44,45^ With regard to inducing antibodies that cross-react with the HA of viruses belonging to heterologous clades of A(H5N1) viruses, MVA-H5(A/Vietnam/04) performed superior compared to MVA expressing HA of influenza A viruses A/Indonesia/5/05 (clade 2.1), A/HK/156/97 (clade 0), A/Turkey/Turkey/1/05 (clade 2.2), A/Chicken/Egypt/3/06 (clade 2.2) or A/Anhui/1/05 (clade 2.3).^45,46^ Finally, immunization with MVA-H5(A/Vietnam/04) protected macaques from challenge infection with the both homologous virus and clade 2.1 virus A/Indonesia/5/05.^47^ This favorable outcome justified testing of MVA-H5(A/Vietnam/04) in a phase 1/2a clinical trial that showed that this vaccine candidate was immunogenic in man. VN antibodies were induced against the homologous strain that cross-reacted not only with heterologous A(H5N1) viruses, but even with a newly emerging A(H5N8) avian influenza virus.^48,49^ The vaccine was well tolerated and serious adverse events were not observed. Collectively, these data show that MVA-based A(H5) vaccines are safe and able to induce cross-clade specific antibodies at levels that are considered protective.

In addition, MVAs expressing HA genes derived from A(H3) and A(H7) influenza viruses have been tested in animal models. A MVA vaccine expressing the HA gene of an A(H3N8) equine influenza virus (A/equine/Kentucky/1/81) was tested in ponies and was shown to induce antibody and T-cell responses, and afforded protection from disease caused by challenge infection with the homologous virus.^50^ Recently, avian influenza viruses of the A(H7N9) subtype have caused frequent infection of humans, especially in Southeast China, often with fatal outcome. A candidate MVA-based vaccine expressing the HA gene of one of these viruses (A/Shanghai/2/13) was constructed and tested in ferrets. It was shown that immunization with MVA-H7(A/Shanghai/2/13) induced VN antibodies and offered partial protection against challenge infection with a closely-related A(H7N9) virus.^51^

#### MVA-NP, MVA-M1, MVA-NA and MVA-PB1 vaccines

Whereas HA is regarded as the antigen of choice for the induction of VN antibody responses, the more conserved internal NP and Matrix 1 (M1) protein are often regarded as antigens of choice for the induction of T-cell responses, in particular CD8^+^ CTL responses. In general, the HA-based vaccines induce a relatively narrow antibody response, which afford little cross-protective immunity to viruses of other subtypes. In contrast, the use of more conserved influenza virus proteins in vaccines may lead to the induction of T-cells directed to epitopes that are shared by influenza viruses of various subtypes and may confer broader protection. For the induction of broadly-protective immunity, MVA-M1, MVA-NA, MVA-PB1 and MVA-NP vaccines were constructed and tested in animal models. Of note, vaccines that aim at the induction of T cell immunity exclusively will not afford sterile immunity, because initial infection and some degree of virus replication cannot be prevented. However, CTL recognize and eliminate virus-infected cells and thereby contribute to viral clearance and recovery.

Hessel *et al* constructed a recombinant MVA expressing the NP gene of an A(H5N1) virus (MVA-NP(A/Vietnam/1203/04)). Immunization of mice with this vaccine candidate not only protected animals from infection with the homologous virus, but also against infection with viruses of the A(H7N1) and A(H9N2) subtype.^52^ However, a similar MVA-NP(A/Vietnam/1203/04) construct failed to induce protective immunity in macaques.^41^ The use of MVA expressing the M1 or PB1 gene from an A(H5N1) virus also failed to induce protective immunity against infection.^52^ MVA expressing the NP gene of an equine A(H3N8) virus (MVA-NP(A/Equine/Kentucky/1/81)) offered ponies partial protection from infection, but only after initial priming with a DNA vaccine expressing the same antigen.^50^ MVA expressing the NA gene from an H1N1pdm09 virus afforded partial protection against H1N1pdm09 challenge infection.^39^

#### Simultaneous delivery of multiple influenza virus antigens by MVA

In order to elicit both protective antibody and T-cell responses simultaneously, MVA expressing both the HA and NP gene have been constructed. In mouse studies, the use of a MVA-H1+NP(A/PR/8/34) vaccine induced virus-specific antibodies and T-cell responses and fully protected mice from infection with the homologous virus and partially protected against infection with a unrelated A(H3N2) virus.^37,53^ Similarly, an MVA-H1+NP was constructed that contained the HA gene of an H1N1pdm09 and the NP gene of a A(H5N1, A/Vietnam/1203/04) virus. In mice, vaccination afforded complete protection from infection with the homologous A(H1N1) and A(H5N1) strains and partial protection from infection with a virus of the A(H3N2) subtype.^54^ A similar MVA-H1+NP construct was tested in macaques, full protection was observed against infection with H1N1pdm09 virus. When the HA gene was replaced by that of an A(H5N1) virus, macaques were only partially protected from infection with the H1N1pdm09 virus.^41^ An MVA simultaneously expressing the HA and NA genes of A(H5N1) virus A/Vietnam/1203/04 and the IL-15 gene was tested in mice and was shown to afford protection against infection with the A(H5N1) virus.^55^

A MVA vaccine designed to induce T-cell responses, expressing the NP and M1 genes of an A(H3N2) virus was extensively tested; first in animal models and then in clinical trials. Immunization of mice with MVA-NP+M1(A/Panama/2007/09) afforded protection against development of severe disease after infection with H1N1pdm09 and A(H3N2) influenza viruses, but not against infection with the mouse-adapted A(H1N1) virus A/PR/8/34. In these studies the recombinant MVA was given in adjunct with recombinant adenovirus expressing the same genes. With the same vaccination strategy chickens were protected from infection with a A(H7N7) virus. Thus with recombinant MVA expressing the conserved NP and M1 genes simultaneously broad immune responses are induced that protect against various subtypes of influenza A virus.^56-59^ This vaccination regimen proved immunogenic in pigs, but the protective potential was not tested in this species.^58^ An MVA-NP+M1 vaccine was subsequently tested in phase 1/2a clinical trials and was shown to induce virus-specific CD8^+^ T-cells in humans and protect from experimental challenge infection with an A(H3N2) virus.^58,60,61^ Furthermore, this vaccination regimen was again shown to be safe and immunogenic in the elderly,^62^ and could be co-administered with TIV.^63^

#### Universal influenza vaccines on basis of MVA

Because of the variable nature of influenza viruses and the extensive antigenic variation they display, the availability of so-called universal vaccines is highly desirable. Some of the MVAs that were discussed above induced cross-reactive immune responses, in particular based on the induction of virus-specific T cells. Commonly unmodified antigens are being used, but currently various modifications of viral proteins are being tested in order to design vaccines that induce broader immunity. Some of these modified influenza viral antigens are now being expressed by recombinant viral vectors, including MVA, and are being tested in animal models.

Recently, Kamlangdee *et al* generated an MVA-H5Mosaic construct, an MVA expressing a computationally generated mosaic A(H5) gene reflecting gene sequences of 2,145 A(H5N1) virus isolates. The MVA-H5Mosaic vaccine was capable of inducing antibodies that reacted with A(H5N1) viruses from all clades, but that did not cross-react with viruses of other HA subtypes. Interestingly, immunized mice were not only protected from infection with various A(H5N1) viruses, but also from infection with A/PR/8/34, a virus of the A(H1N1) subtype. Protection from challenge infection with an A(H3N2) could not be achieved.^64^

Another vaccine approach of interest is the design of vaccines that can induce broadly-reactive and VN antibodies to the conserved stalk region of the HA molecule.^65^ A single study has performed vaccination challenge experiments with MVA expressing the HA-stalk, either alone or in combination with other antigens.^52^ The use of MVA-H5Stalk(A/Vietnam/1203/04) alone or in combination with an MVA expressing 4 M2e repeats from A(H5N1), A(H9N2), A(H7N2) and A(H1N1) did not afford protective immunity. However, when the MVA-H5Stalk (with or without the MVA expressing M2e) construct was combined with an MVA expressing an NP gene (MVA-NP(A/Vietnam/1203/04)), mice were protected from infection with A(H5N1), A(H7N1) and A(H9N2) viruses.^52^ Co-administration of an NP expressing vaccine seemed essential in achieving cross-protection in these studies, corresponding to the induction of broadly-reactive virus-specific CD4^+^ and CD8^+^ T-cells.

Thus, it was shown that influenza virus antigens can be modified to optimize induction of (cross-reactive) immune responses and these modified viral antigens can readily be expressed by recombinant MVA vectors. Thus MVA provides an ideal platform for the expression of modified influenza viral proteins, smartly designed for the induction of broadly protective immunity.

#### NYVAC, Raccoonpox, Canarypox and Fowlpox vectors

Next to MVA, other attenuated poxviruses have been used as viral vectors for the development of candidate influenza vaccines. Immunization of chickens with recombinant NYVAC, a VV strain highly attenuated by deletion of 18 open reading frames from the viral genome,^66^ expressing the HA gene of an avian A(H5N1) virus (NYVAC-H5(A/Chicken/Indonesia/7/04)), afforded protection against infection with a heterologous A(H5N2) virus.^67^ Recombinant Raccoonpox (RCN) viruses expressing the HA, NA or NP genes of A(H5N1) influenza virus A/Vietnam/1203/04 were constructed and evaluated for their protective capacity in mice. Interestingly, protection from A(H5N1) challenge infection could be demonstrated but was dependent on the route of administration. Intra-dermal immunization with RCN-HA afforded protection, protection was also observed with RCN-NA but only after intra-nasal administration.^68,69^

Canarypox (CNPV) is a host-restricted member of the poxvirus family that is unable to produce infectious progeny virus in mammalian cells, making it potentially safe as a viral vector for human use. At present, recombinant CNPV are used as influenza vector vaccines for vaccination of horses against equine influenza of the A(H3N8) subtype. Recombinant CNPV vaccines expressing HA genes of various A(H3N8) strains were immunogenic in horses and protected against infection with virus of this subtype.^70-75^ Furthermore, maternal antibodies induced by CNPV-HA were passively transferred to foals of vaccinated pregnant horses.^76^ In addition, CNPV was also used as a vaccine vector to protect pigs and cats from avian influenza virus of the A(H5N1) subtype. Pigs were vaccinated with CNPV-HA(A/Chicken/Indonesia/7/03) and proved to be protected from infection with an unrelated virus of the A(H5N2) subtype.^67^ In cats, the same vaccine afforded protection from infection with various A(H5N1) viruses.^77^

Fowlpox virus (FPV) is the causative agent of an economically important disease of chickens. However, several attenuated FPV vector vaccines expressing influenza virus antigens are currently licensed as A(H5N1) influenza vaccines for use in poultry. Initial studies performed in 1998 showed that FPV-HA vaccines could protect chickens and turkeys from experimental challenge infections.^78^ Comparable to MVA, it was recently shown that FPV as a vector efficiently induces both B- and T-cell responses in chickens,^79^ however it has also been shown that pre-existing immunity to FPV or influenza antigens could pose a potential problem when using FPV-based influenza vaccines.^80^

An experimental FPV-based A(H5) vaccine, expressing the HA gene of an A(H5N8) virus (A/Turkey/Ireland/1378/83) was extensively tested in chickens and was shown to offer protection against infection with the homologous virus, but also against viruses of the A(H5N1), A(H5N2), A(H5N3), A(H5N8) and A(H5N9) subtypes.^81-83^ Various other FPV-H5 vaccines were constructed and tested in chickens and ducks and proved to afford protection against infection with homologous and heterologous viruses.^84-89^ Furthermore, recombinant FPV vaccines were generated expressing the HA genes of A(H1), A(H7) and A(H9) viruses. An FPV-H1(A/PR/8/34) failed to protect chickens from infection with an A(H7N7) virus,^90^ but vaccination with recombinant FPV expressing the homologous HA gene afforded full protection.^90,91^ Similar results were obtained with a recombinant FPV vaccine expressing the HA gene of avian A(H9N2) influenza virus.^92,93^ Interestingly, co-expression of IL-6 or IL-18 genes improved immunogenicity of FPV-HA vaccines in chickens and ducks.^85,89^

Recombinant FPV vaccines expressing influenza virus NP and NA genes were tested as well. A recombinant FPV expressing NP failed to protect chickens from infection. However, a FPV-based vaccine expressing both the HA and NP genes simultaneously protected chickens completely from homologous challenge infection.^94^ Notably, recombinant FPV expressing both the HA and NA gene of A(H5N1) virus A/Goose/Guangdong/3/96 protected chickens completely not only against infection with the homologous virus, but also against infection with a virus of another subtype, namely A(H7N1).^95,96^ Although FPV vector vaccines expressing influenza viral antigens rarely have been used in non-avian species, FPV-HA was capable of inducing antibody responses in cats ^97^ and afforded protection in pigs when challenged with a low-pathogenic A(H5N2) influenza virus.^67^

### Alphavirus vectors

Alphaviruses are single-stranded RNA viruses with a positive sense genome of the *Togaviridae* family. Several alphaviruses are being developed as vaccine vectors, including semliki forest virus (SFV), sindbis virus (SIN) and Venezuelan equine encephalitis (VEE). These vectors often are replication deficient replicons that do not encode viral structural proteins, as these regions of the genome are replaced by transgenes of interest. Viral RNAs are self-replicating and are capable of transgene expression at high levels.^98^ As an added advantage, when using alphavirus replicons pre-existing immunity to the vector should not pose a problem and multiple sequential vaccinations are a possibility.^99-102^ Furthermore, VEE is an appealing vaccine vector, as it was previously shown that VEE mainly targets antigen-presenting cells in the lymphoid tissues and therefore primes rapid and robust immune responses.^103^

SIN, SFV and VEE have all been tested as influenza vaccine vectors (Table 2). An SFV vaccine expressing the HA and NP genes could protect mice from infection with A(H1N1) virus;^99^ the same held true for a SIN replicon expressing either the HA gene or an immunodominant CD8^+^ T-cell NP epitope.^104,105^ VEE was more extensively evaluated as an influenza vaccine vector, VEE-H1(A/PR/8/34) vaccination protected mice from infection with homologous virus,^101^ even in aged animals.^106^ Immunization of chickens with VEE expressing the HA gene of A(H5N1) virus A/HK/156/97 also afforded full protection against this virus.^107^ Finally, vaccination of pigs has been studied using different VEE constructs, expressing HA genes of the A(H1) and A(H3) subtypes and an NP gene of an A(H3N2) swine influenza virus. In all cases antibodies were induced by vaccination, and pigs could at least be partially protected from infection with the homologous virus.^108-112^ Heterosubtypic immunity could be induced when pigs were vaccinated with the VEE-NP construct,^112^ however VEE-based vaccines performed poorly in the presence of maternal antibodies.^108^

**Table 2.** Overview of vector-based influenza vaccines.

Vector	Model	Antigen	Modification	Subtype	Reference
					
Alphavirus		HA	n/a	H1N1	^100,103,105^
		NP	CD8 epitope	n/a	^104^
		HA/NP	n/a	H1N1	^98^
					
		HA	n/a	H1N1, H3N2	^107-111^
		NP	n/a	H3N2	^111^
		HA/NP	n/a	H3N2	^107^
					
		HA	n/a	H5N1	^106^
Herpesvirus		HA	n/a	H1N1, H3N2, H3N8	^130,131,133^
					
		HA	n/a	H1N1	^129,134^
		HA/NA	n/a	H1N1	^128^
					
		HA	n/a	H5N1, H5N2, H7N1	^115,117-126^
		NA	n/a	H5N1	^120^
		HA/NA	n/a	H5N1	^120^
					
		HA	n/a	H5, H5N1	^114^
					
		HA	n/a	H3N8	^131^
					
		HA	n/a	H3N8	^132^
VSV		HA	n/a	H1N1, H5N1	^136,139,143,145^
		HA	Chimeric	cH5/1, cH9/1	^140^
		NP	n/a	H1N1	^139^
		HA/NP	n/a	H1N1	^139^
		HA/NA	n/a	H1N1, H5N1	^140^
					
		HA	n/a	H5N1, H7N1	^142^
					
		HA	n/a	H5N1	^144^
NDV		HA	n/a	H1N1, H5N1	^147,158,159,167^
		HA	Soluble	H5N1	^158^
					
		HA	n/a	H5N1, H5N2, H6, H7, H7N2, H7N9, H9N2	^149,150,152,153,155-166^
		HA	Soluble	H5N1	^158^
		HA	Ectodomain	H5N1	^151^
		HA/NA	n/a	H5N1	^155^
					
		HA	n/a	H6	^165^
					
		HA	n/a	H5N1, H5N8	^149,154^
					
		HA	n/a	H5N1	^168,169^
Baculo		HA	n/a	H1N1, H5N1, H6N8, H7N9, H9N2	^171-178^
PIV-5		HA	n/a	H3N2, H5N1, H7N9	^180-182^
		NP	n/a	H5N1	^182^
					
		HA	n/a	H7N9	^181^
		HA/NP	n/a	H7N9	^181^
					
		HA	n/a	H3N2	^179^

A VEE-based candidate vaccine against cytomegalovirus (CMV) has been tested in clinical trials, was shown to be immunogenic, well tolerated and safe in humans.^113^ Clinical trials with alphaviruses-based vector influenza vaccines have not yet been conducted, however since these replicons are potentially efficacious (even in the face of pre-existing immunity) and safe, they hold promise as influenza vector vaccines.

### Herpes virus vectors

Several recombinant herpes viruses have been tested in animal models as candidate influenza vaccines (Table 2). Duck enteritis virus (DEV), an alphaherpesvirus and the causative agent of duck plague, has caused fatal infections in many species of waterfowl. However, deletion of glycoprotein C (gC) leads to an attenuated DEV that may be exploited for the development of vectored vaccines. Construction of the first DEV-based candidate A(H5N1) influenza vaccine was initially described in 2011.^114^ Subsequently, it was shown that DEV encoding different HAs of A(H5N1) viruses could protect chickens and ducks from lethal infections with these viruses^115-117^ and was capable of inducing both humoral and cellular immune responses.^118^

Infectious laryngotracheitis virus (ILTV), another alphaherpesvirus that causes disease in mainly poultry, has been tested as an influenza vaccine for poultry. Attenuated strains of ILTV expressing HA genes obtained from various A(H5N1) and A(H5N2) influenza virus strains were generated and shown to offer protection against these viruses.^119,120^ However, ILTV should be attenuated sufficiently, as pathogenicity caused by the vector was still observed in a single study.^119^ ILTV vaccines expressing the NA gene were also generated but were poorly immunogenic; co-expression of HA genes was always required to obtain protective immunity.^121^

Another alphaherpesvirus of poultry, turkey herpesvirus (HVT) has also been extensively studied as influenza vaccine vector in chickens. HVT encoding the HA gene of an A(H5N1) virus afforded protection from infection with various A(H5N1) viruses.^122-124^ Similarly, a recombinant HVT-H7 vaccine protected chickens against infection with the homologous A(H7N1) virus.^125^ Since chickens are often vaccinated at very young age (1 day after birth), maternal antibodies against the vector or against the protein encoded by the transgene could influence vaccine efficacy. Interestingly, HVT was shown to be immunogenic even in the presence of these maternal antibodies.^126^ Marek's disease virus (MDV), an alphaherpesvirus closely related to HVT, was shown to be an effective vaccine vector against A(H5N1) virus and even performed better than HVT in a side-by-side comparison in chickens.^127^

Pseudorabiesvirus (PrV) is an alphaherpesvirus of pigs and attenuated strains of PrV have been generated and used for control of Aujeszky's disease in pigs.^128^ Attenuated PrV expressing foreign antigens were generated and are attractive as bivalent vaccines for pigs. The use of PrV-H1 influenza vaccines partially protected pigs from H1N1pdm09 virus infection. Recombinant PrV expressing the NA gene derived from a swine A(H1N1) influenza virus only protected pigs to a limited extent.^129,130^ A recombinant PrV expressing HA from a swine A(H3N2) virus was tested in mice and induced protective immunity against infection with heterologous A(H3N2) virus.^131^

An attenuated strain of equine herpesvirus (EHV-1), an alphaherpesvirus that infects horses, has an impressive safety record in horses and other mammalian species and therefore should be considered an attractive vaccine vector. The HA gene from an equine A(H3N8) influenza virus was cloned into EHV-1 and could induce antibody responses that react with multiple A(H3N8) strains in mice and horses.^132,133^ Interestingly, the protective efficacy of these vaccines was only assessed in dogs, which upon vaccination were partially protected from infection with a canine A(H3N8) influenza virus strain.^132^ In addition, recombinant EHV-1 were constructed that express the HA gene derived from H1N1pdm09 virus. This vaccine candidate afforded mice complete and pigs partial protection from infection.^134,135^

Herpes viruses encoding influenza virus antigens have mainly been tested as candidate vaccines for poultry in which their protective effectiveness was confirmed. Like other DNA viruses, herpes viruses have a relatively large genome and antigens of interest can easily be cloned into multiple insertion sites. Although an advantage, it also complicates characterization of the vector because the insertion site of the transgene may influence its immunogenicity.^122^

### Vesicular stomatitis virus vectors

Vesicular Stomatitis Virus (VSV) is a rhabdovirus and has a negative sense RNA genome. Candidate influenza vaccines based on VSV have been constructed and have numerous advantages over other vectors. VSV is immunogenic,^136,137^ has a broad tissue tropism and can easily be delivered locally. In contrast to other vectors (like adeno- and poxviruses), there is little evidence for VSV seropositivity in humans, eliminating concerns of pre-existing immunity to the vector. On the other hand, use of VSV as a vector is not without concern: VSV can cause disease in humans^138^ and is known to be neuro-invasive in some species.^139^ Currently, there is no human safety data available for VSV-vectored vaccines and additional experiments are required. An overview of VSV-based influenza vaccines can be found in Table 2.

VSV expressing the HA gene of influenza virus A/WSN/33 (H1N1) proved to be immunogenic in mice and protected the animals from challenge infection.^137^ Since VSV also proved to be pathogenic in mice, most studies with VSV as vector relied on recombinant attenuated VSV viruses with a truncated or deficient G protein. In similar experiments, mice could only be partially protected from infection with influenza virus A/PR/8/34 after vaccination with VSV expressing the corresponding HA gene, whereas expression of only the NP failed to afford protection. However, combination of the HA and NP constructs resulted in full protection from infection.^140^ Furthermore, a VSV expressing the HA gene of influenza virus A/Vietnam/1203/04 (H5N1) and the NA gene of influenza virus A/PR/8/34 (H1N1) completely protected mice from infection with a 6:2 reassortant A(H5N1) virus (HA and NA from A/Vietnam/1203/04).^141^ VSV-H7(A/FPV/Rostock/34) expressing the HA gene of an A(H7N1) virus afforded chickens protection from developing disease after caused by a virus of the same subtype.^142^

VSV-based candidate A(H5N1) vaccines were constructed and tested in chickens, mice and macaques. Immunization with VSV expressing the HA gene of 2 different avian A(H5N1) viruses completely protected chickens from A(H5N1) virus challenge.^143^ Also in mice and macaques VSV-based A(H5N1) vaccines proved to be immunogenic^144-146^ and mice immunized with a recombinant VSV expressing the HA gene of a clade 0 A(H5N1) virus were protected from infection with viruses of the same clade and those of clade 1.^144,146^

VSV has also been used to construct vaccines that aim at the induction of broadly reactive HA-stalk specific antibodies as a universal influenza vaccine approach. As suggested previously, repeated vaccination with various chimeric HA molecules can boost the induction of stalk-specific antibody responses.^147^ Therefore, mice were primed with a recombinant VSV expressing an HA gene with the stalk region of influenza virus A/PR/8/34 and the globular head domain of an A(H9N2) virus. Subsequently mice were boosted with a VSV, expressing an HA gene with the same stalk but with the head domain of an A(H5N1) virus (VSV-cH5/1). As expected, mice could be protected from infection with influenza virus A/PR/8/34 by this vaccination regimen.^141^ Priming with VSV encoding the full-length HA gene of A/PR/8/34 (H1N1) followed by boost with the VSV-cH9/1 construct also afforded protection against infection with a virus of the A(H5N1) subtype.^141^ Interestingly, in both experiments it was shown that intra-nasal prime-boost regimens performed better than IM vaccination.

### Newcastle disease virus vectors

Newcastle disease virus (NDV) is a single-stranded negative sense RNA paramyxovirus that causes disease in poultry. NDV has several favorable properties as a vaccine vector; no pre-existing immunity in humans exists, NDV can easily be attenuated and reverse genetics systems to rescue recombinant NDV are in place. Thus far, NDV has been extensively characterized as an influenza vaccine vector in poultry, where it serves as a bivalent vaccine capable of inducing immunity against both NDV and influenza virus. As an added advantage, NDV is easily administered to poultry through nasal spray, drinking water or ocular drops. An overview of NDV-based influenza vaccines can be found in Table 2.

The first study using NDV as a vaccine vector for influenza was NDV-H1, that expressed the HA gene of influenza virus A/WSN/1933. Complete protection of mice against homologous challenge infection was achieved, demonstrating that NDV can be used as an influenza vaccine vector.^148^ Consequently, a recombinant NDV expressing HA genes of A(H5N1) viruses has been licensed as a poultry vaccine in some countries and was shown to have a protective effect against challenge infection with A(H5N1) viruses in chickens and ducks in various studies.^149-163^ The NDV based A(H5N1) vaccine offered only partial cross-clade protection, but was immunogenic in the presence of maternal antibodies.^162,163^ Expression or co-expression of NA by NDV did not improve immunogenicity in chickens.^156^ Also NDVs expressing the HA genes of A(H6), A(H7) and A(H9) subtypes were tested in poultry. Although most challenge viruses were low-pathogenic, a reduction or complete abrogation of virus shedding could be obtained after inoculation with the respective homologous viruses.^161,164-167^

To develop NDV-based vaccines for use in humans, their performance has also been tested in mammalian species. In mice, protective immunity against A(H5N1) viruses was induced after vaccination with NDV expressing the homologous HA gene.^159,160^ In one single study, cross-reactive cellular immune responses against A(H1N1) viruses were observed after vaccination with a NDV-H5 construct.^168^ The immunogenicity of recombinant NDV expressing the HA and NA genes of influenza virus A/Vietnam/1203/04 (H5N1) was tested in non-human primates. Both constructs induced VN and local IgA antibody responses and afforded protection from A(H5N1) challenge infection.^169,170^ Small numbers of clinical trials have been performed with NDV, which showed that the vector is well tolerated.

### Baculovirus vectors

Baculoviruses are extensively used as tool to express and produce influenza virus proteins. Currently, a recombinant HA protein vaccine produced in baculoviruses was approved for human use in the United States. However, baculoviruses have also been explored as live vaccine vectors. Since baculoviruses can readily be manipulated to express foreign antigens and can infect mammalian cells without causing cytopathic effect they are potentially promising vaccine vectors for influenza (Table 2).^171^

Initially, it was reported that vaccination with recombinant baculovirus expressing the HA gene of influenza virus could induce complete protection from homologous challenge infection.^172^ Interestingly, in this study the control group that received an ‘empty’ baculovirus, not expressing the HA gene, was also protected from challenge infection. Potentially the induction of strong non-specific innate immune responses by vaccination with baculovirus was responsible. Subsequently, several baculoviruses expressing the HA genes of various A(H5N1) influenza viruses were tested in mice and afforded protection against infection with both homologous viruses and A(H5N1) viruses from different clades.^173,174^ Bivalent baculovirus vaccines, expressing 2 different HA genes from A(H5) viruses simultaneously, were also successful in affording cross-clade immunity.^175,176^ Finally, recombinant baculoviruses expressing HA genes of A(H6), A(H7) and A(H9) influenza viruses were capable of inducing protective immunity against infection with homologous viruses in mice.^177-179^

Although recombinant baculovirus vector vaccines were tested in mice, efficacy data in other animal models is still lacking. Short-term production of baculovirus-based influenza virus vaccines for use in clinical trials is therefore not likely.

### Parainfluenza virus 5 vectors

Parainfluenza virus 5 (PIV-5) is, like NDV, a negative sense RNA paramyxovirus that is only recently being explored as an influenza virus vaccine vector (Table 2). Favorable properties of PIV-5 as a vector include: broad tissue and cell tropism, no clinical disease in humans and availability of reverse genetics systems. Although PIV-5 does not cause disease in humans, PIV-5 has been associated with ‘kennel cough’ in dogs.^180^

In an initial study, vaccination with PIV-5 expressing the HA of an A(H3) virus afforded protection against homologous challenge infection.^181^ PIV-5 expressing the HA genes of A/Vietnam/1203/04 (H5N1) and A/Anhui/1/13 (H7N9) also completely protected mice from infection with the homologous influenza virus.^182,183^ PIV-5 expressing an internal protein of influenza virus, in this case the NP gene of A/Vietnam/1203/04, was constructed, but could only partially protect mice from homologous challenge infection. Interestingly, PIV-5 expressing the same NP gene completely protected mice from a heterologous challenge infection with A(H1N1), cellular immune responses targeting NP were the responsible correlate of protection.^183^ Similar results were obtained with recombinant PIV-5-NP(A/Anhui/1/13) in guinea pigs challenged with a homologous A(H7N9) influenza virus.^182^

PIV-5 has been evaluated in mice and guinea pigs, but was not tested as an candidate influenza vaccine in other animal models. Furthermore, clinical trials in humans have not been performed with PIV-5 yet, so safety and efficacy data is therefore not available. Finally, little is known about pre-existing immunity to the vector in humans. However, in dogs, a PIV-5 vector vaccine expressing the HA gene of influenza virus could still induce robust antibody responses in the presence of PIV-5-specific immunity.^180^ It remains to be determined whether PIV-5 is safe and immunogenic when used in humans.

### Adenovirus vectors

Recombinant adenoviruses (rAd) have attractive properties to serve as vaccine vectors: high titer stocks can be grown, genes of interest can easily be inserted into the stable viral genome, long-term storage at 4 degrees is possible and rAd infects a variety of hosts, tissues and cell types.^184^ Furthermore, rAd can even induce robust immune responses when administered orally or intra-nasally, potentially bypassing pre-existing immunity against the vector.^184^ Finally, even replication-deficient rAd are known to be immunogenic; adenovirus 5 (Ad5) is a replication-deficient vector that has been evaluated for gene delivery, anti-cancer therapy and as an infectious disease vaccine. An overview of adenovirus-based influenza vaccines can be found in Table 3.

**Table 3.** Overview of adenovirus-based influenza vaccines.

Vector	Model	Antigen	Modification	Subtype	Reference
					
Adenoviruses		HA	n/a	H3N2, H5N1, H7N7, H9N2	^191,193-195,199,201-204,206^
		HA	Soluble head	H1N1	^198^
		HA	Glycan shielded	H5N1	^207^
		NP	n/a	H1N1, H5N1	^192,208,209,214^
		M2	Consensus	n/a	^180^
		HA/NP	n/a	H5N1, H7N7, H9N2	^206^
		HA/NA	n/a	H1N1	^211^
		NP/M2	n/a	H1N1	^216^
		NP/M2	Consensus	H1N1	^210^
		HA/NA/M1	n/a	H1N1, H5N!	^213^
		NP/M1/M2	miRNAs	H1N1	^211^
					
		HA	n/a	H5N1	^203,215^
		NP	n/a	H5N1	^215^
		M2	n/a	H5N1	^215^
		HA/NA	Consensus	H1N1	^212^
		NP/M2	n/a	H1N1, H5N1	^215,216^
		HA/NP/M2	n/a	H5N1	^215^
					
		HA	n/a	H1N1, H3N2	^196,200^
		NP	n/a	H3N2	^196^
		HA/NP	n/a	H3N2	^196^
					
		HA	n/a	H5N1, H7N3	^202,205^
					
		HA	n/a	H1N1, H5N1	^187,188,190^
		NP/M1	n/a	H3N2	^189^
AAV		HA	n/a	H1N1	^222^
		HA	Broadly neutralizing ab	n/a	^223,224^
		NP	n/a	H1N1	^222^
		M1	n/a	H1N1	^222^
		HA/NP/M1	n/a	H1N1	^222^
					
		HA	Broadly neutralizing ab	n/a	^224^

A live adenovirus vaccine that contains 2 different serotypes is already in use for vaccination of humans for decades,^185^ indicating that adenoviruses are safe and immunogenic in humans. However, continuation of clinical trials with rAd5 is currently hampered by 2 trial failures: one death was reported after intra-venous rAd5 administration,^186^ another study showed increased risk of acquiring HIV-1 infection after vaccination with rAd5 expressing HIV-1 genes gag, pol and nef.^187^ However, recombinant adenovirus expressing the HA gene of influenza virus A/PR/8/34 proteins proved to be safe and immunogenic in humans, inducing mainly a robust antibody response.^188^ A more recent trial in humans with rAd4 expressing the HA gene of an A(H5N1) influenza virus reported enhanced immune responses after co-administration with an HA protein vaccine in the absence of serious adverse events.^189^ Finally, a rAd expressing the NP and M1 genes of influenza virus and a rAd5 expressing the HA gene of an A(H1) virus and co-expressing dsRNA as adjuvant were safe and immunogenic in humans.^190,191^

In addition to the 2 discussed trial failures, a second drawback for the use of rAd in humans is the potential of pre-existing immunity against adenovirus interfering with vaccine efficacy. Currently, as alternatives, non-human adenoviruses ^190,192-194^ and low-prevalent adenoviruses ^195^ are being explored as novel vaccine vectors.

#### Ad-HA vaccines

Adenoviruses expressing HA genes of a number of different subtypes (A(H1, H3, H5, H7 and H9)) have been tested in various animal models. In the first study with rAd5, a vaccine that expressed the HA gene of an A(H3N2) influenza virus of swine-origin protected mice from challenge infection with a heterologous A(H3N2) virus.^196^ A rAd expressing the HA gene of a different A(H3) virus was shown to be efficacious in pigs,^197^ even in the presence of maternal antibodies.^198^ Adenovirus vaccines expressing the HA gene of A/PR/8/34, completely protected mice from homologous challenge infection.^195,199,200^ Pigs could also be protected from A/PR/8/34 virus infection by vaccination with rAd5 expressing the HA gene from the H1N1pdm09 virus A/Cal/04/09. Interestingly, pigs were also partially protected by vaccination with this construct from infection with a heterologous A(H1N2) virus.^201^ A rAd expressing the HA gene from A(H5N1), protected mice, chickens and ferrets from infection with the homologous virus,^202-204^ when the HA gene of A/HK/156/97 was introduced into rAd cross-clade protection was reported.^205^ The rAd expressing the HA gene of an A(H7) virus was immunogenic in chickens and capable of protecting chickens from homologous challenge infection.^206^ In 2013, a comprehensive study testing rAd5 vectors expressing the HA genes from avian viruses of the A(H5), A(H7) and A(H9) subtype (and combinations thereof) showed that mice could be protected from homologous challenge infection. Heterosubtypic immunite was never observed, however it was shown that simultaneous vaccination with 5 different rAd5-HA vaccines was feasible and protected from challenge infection with viruses of all subtypes under investigation.^207^

Comparable to expressing modified influenza antigens in other vectors with the goal of inducing universal influenza immunity (*i.e.* ‘headless’ HA, chimeric HA, consensus sequences), a rAd expressing a modified HA gene was constructed. This HA gene was modified to shield the immunodominant head region by glycans to re-direct the immune response from the HA head region to target the more conserved stalk region and afford broad protection. Indeed, a rAd expressing the HA gene of an A(H5) influenza virus, either wildtype or glycosylated, afforded cross-clade protection in mice, the glycosylated HA performed better than its wildtype counterpart.^208^ Heterosubtypic immunity with these glycan-shielded constructs has not been reported yet.

#### Ad-NP, Ad-M1, Ad-M2 and Ad-NA vaccines

Different vaccination regimens with rAd constructs expressing the NP gene were tested in animal models with some reports of heterosubtypic immunity. A rAdC7 expressing the NP gene of A/PR/8/34 could partially protect mice from infection with some - but not all - influenza viruses of the A(H5N1) subtype.^193^ Vice versa, rAd expressing the NP gene of an avian A(H5N1) virus completely protected mice from infection with A(H1N1) virus.^209^ However, vaccination of pigs with a comparable rAd5-NP construct could not afford protection from homologous challenge infection, whereas addition of a rAd5-HA construct to the vaccine cocktail completely restored the protective capacities.^197^

Vaccination of mice with a combination rAd5 vaccine, including constructs expressing both the NP and M2 genes, protected mice from homologous challenge infection.^210^ A rAd5 expressing an M2 consensus sequence could even afford protection from infection with various A(H1N1) influenza viruses^181^ and abrogated contact transmission to sentinel mice.^211^ When both NP and M1 consensus gene sequences were expressed by rAd, vaccination led to partial immunity to A(H1N1) virus infection in mice. A similar rAd vaccine, expressing the NP and M1 genes from an A(H3N2) virus, induced T-cell responses and proved to be safe in humans.^190^ Recently, a novel approach was tested when rAdC68 was constructed to express miRNAs that target NP, M1 and M2 RNA from A/PR/8/34 influenza virus, and these constructs protected mice from A(H1N1), A(H5N1) and A(H9N2) challenge infection.^212^

A bivalent rAd5 vaccine, expressing the HA and NA consensus gene sequences of multiple H1N1pdm09 viruses protected both mice and ferrets from infection with H1N1pdm09 influenza virus.^213^ A trivalent vaccine expressing the M1 gene in addition to the HA and NA genes from either 1918 pandemic A(H1N1) or avian influenza A(H5N1) protected mice from challenge infection with A(H5N1) viruses from different clades. Taking this one step further, pentavalent vaccines that expressed the HA, NA and M1 genes from avian A(H5N1) and the HA and NA genes from 1918 pandemic A(H1N1) performed superior in inducing protection from A(H5N1) infection.^214^

#### Adenovirus heterologous prime boost regimens

Recombinant Ad5 was extensively tested in heterologous prime boost vaccination regimens, in which animals were primed with DNA encoding the HA, NP and/or M2 genes and subsequently boosted with rAd5 expressing the same proteins. DNA priming followed by rAd5-NP(A/PR/8/34) boost vaccination completely protected mice from homologous challenge infection and afforded heterosubtypic immunity upon infection with A(H3N2) and some A(H5N1) influenza strains.^215^ On the contrary, ferrets could not be protected from A(H5N1) infection by this vaccination regimen.^216^ Similar negative results were obtained in ferrets with rAd constructs expressing the M2 gene.^216^ In heterologous prime boost regimens where DNA vaccination was followed by vaccination with a bivalent rAd5 construct expressing both the M2 and NP genes, mice were completely protected from infection with A(H1N1) and A(H5N1),^217^ but conflicting results were again obtained in ferrets.^216,217^ Recombinant Ad5 expressing the HA gene was always protective in ferrets when a DNA prime followed by rAd5 boost regimen was used, inducing protection against infection with the homologous virus in all cases.^216^

### Adeno-associated virus vectors

Adeno-associated virus (AAV) is a parvovirus that is replication-deficient in humans. Like adenovirus, AAV has a broad cell, tissue and host tropism and therefore is a potential good vector vaccine.^218^ However, drawbacks of using AAV include: limited capacity for transgenes, presence of pre-existing immunity in humans and the technical challenge of producing high titer stocks. Initially, AAV was not explored as a vaccine vector as it was considered to be poorly immunogenic, however vaccination studies in mice showed that AAV-2 expressing an HSV-2 glycoprotein was immunogenic and a potent inducer of T-cell and antibody responses,^219^ and currently modifications are being made to AAV to increase immunogenicity.^220^

A limited number of studies evaluating AAV as a vector for influenza vaccination has been performed (Table 3). Initially, an AAV expressing the HA gene or NP gene was shown to be protective in mice.^221,222^ A more recent study tested AAV vaccines expressing the HA, NP or M1 genes of H1N1pdm09 in mice. Whereas AAV-HA afforded full protection from H1N1pdm09 infection, AAV-NP protected mice partially and AAV-M1 did not afford protection. Simultaneous vaccination with all 3 constructs afforded protection from homologous challenge infection.^223^ Recently, in an alternative vaccination approach, AAV was constructed to express a transgene encoding a influenza virus-specific broadly neutralizing antibody. AAV constructs expressing the broadly neutralizing antibody ‘F10’ protected mice from infection with 3 different A(H1N1) strains,^224^ whereas AAV expressing the broadly neutralizing antibody ‘FI6’ protected mice and ferrets from infection with various A(H5N1) and A(H1N1) viruses.^225^

## Conclusions

Viral vectors have potential as novel vaccine candidates in times of pressing need for game-changing vaccines that induce broadly protective immunity against a wide variety of influenza viruses. The major advantage of viral vectors is the possibility of expressing any foreign antigen with or without modification *in vivo*. Since the proteins are expressed in their native confirmation, antibody responses of the desired specificities are induced. In addition, viral vectors allow *de novo* protein synthesis in the cytoplasm of infected cells facilitating endogenous antigen processing and MHC class I presentation of immunogenic peptides, which is a requirement for the efficient induction of virus-specific CD8^+^ T-cell responses. Although all vectors discussed have their own respective advantages and disadvantages, most are replication-deficient in mammalian host cells and are therefore safe for human use, even in immunocompromised individuals. Pre-existing immunity to the vector may pose a problem for some vectors, however there are viral vectors available (like VSV) for which the human population is immunologically naïve. Other vectors (like MVA) proved to be immunogenic even in the presence of pre-existing immunity. For some vector technologies there are some safety concerns, like the use of herpes viruses that persistently infect their hosts and DNA vaccines that might integrate into the host genome. These properties might restrict their applicability as prophylactic vaccines.

As discussed in this review, viral vectors as potential influenza vaccine candidates were not only evaluated in animal models and humans, they were also extensively tested in influenza A virus reservoir species. Vaccination of reservoir species could potentially limit transmission of avian and swine influenza A virus transmission, and therefore limit the zoonotic transmission of these potential (pre-)pandemic viruses to the human host.

In the future, more novel vector-based influenza candidate vaccines will be developed and tested in clinical trials. There is potential for improvement by the modification of viral antigens, like the ‘headless’ or ‘shielded’ HA constructs, to broaden the reactivity of vaccine induced antibodies. In addition to modifying influenza virus antigens, post-translational modifications and modifications to promoter sequences could also alter and improve the immunogenicity.^226,227^ The biggest challenge of taking vector-based vaccines to the market may be obtaining approval from the regulatory authorities. Only when their safety and superiority over existing vaccine formulations have been demonstrated, implementation of these novel vector-based vaccines may be considered.

## Abbreviations

TIV: Trivalent inactivated vaccine
QIV: Quadrivalent inactivated vaccine
LAIV: Live-attenuated influenza vaccine
IM: Intra-muscular
HA: Hemagglutinin
NA: Neuraminidase
CTL: Cytotoxic T-lymphocyte
VV: Vaccinia virus
MVA: Modified vaccinia virus Ankara
BSL-1: Biosafety level 1
NP: Nucleoprotein
VN: Virus neutralizing
HI: Hemagglutination inhibition
M1: Matrix 1
rAd: Recombinant adenovirus
RCN: Raccoonpox virus
CNPV: Canarypox virus
FPV: Fowlpox virus
SFV: Semliki forest virus
SIN: Sindbis virus
VEE: Venezuelan equine encephalitis
CMV: Cytomegalovirus
DEV: Duck enteritis virus
gC: Glycoprotein C
ILTV: Infectious laryngotracheitis virus
HVT: Turkey herpesvirus
MDV: Marek's disease virus
PrV: Pseudorabies virus
EHV-1: Equine herpesvirus
VSV: Vesicular stomatitis virus
NDV: Newcastle disease virus
PIV-5: Parainfluenza virus 5
AAV: Adeno-associated virus
